# pSBVB: A Versatile Simulation Tool To Evaluate Genomic Selection in Polyploid Species

**DOI:** 10.1534/g3.118.200942

**Published:** 2018-12-20

**Authors:** María L. Zingaretti, Amparo Monfort, Miguel Pérez-Enciso

**Affiliations:** * Centre for Research in Agricultural Genomics (CRAG), CSIC-IRTA-UAB-UB Consortium, 08193 Bellaterra, Barcelona, Spain; † IRTA (Institut de Recerca i Tecnologia Agroalimentàries), Barcelona, Spain; ‡ ICREA, Passeig de Lluís Companys 23, 08010 Barcelona, Spain

**Keywords:** Genomic selection, Polyploids, Simulation, pSBVB, Genomic Prediction, GenPred, Shared Data Resources

## Abstract

Genomic Selection (GS) is the procedure whereby molecular information is used to predict complex phenotypes and it is standard in many animal and plant breeding schemes. However, only a small number of studies have been reported in horticultural crops, and in polyploid species in particular. In this paper, we have developed a versatile forward simulation tool, called polyploid Sequence Based Virtual Breeding (pSBVB), to evaluate GS strategies in polyploids; pSBVB is an efficient gene dropping software that can simulate any number of complex phenotypes, allowing a very flexible modeling of phenotypes suited to polyploids. As input, it takes genotype data from the founder population, which can vary from single nucleotide polymorphisms (SNP) chips up to sequence, a list of causal variants for every trait and their heritabilities, and the pedigree. Recombination rates between homeologous chromosomes can be specified, so that both allo- and autopolyploid species can be considered. The program outputs phenotype and genotype data for all individuals in the pedigree. Optionally, it can produce several genomic relationship matrices that consider exact or approximate genotype values. pSBVB can therefore be used to evaluate GS strategies in polyploid species (say varying SNP density, genetic architecture or population size, among other factors), or to optimize experimental designs for association studies. We illustrate pSBVB with SNP data from tetraploid potato and partial sequence data from octoploid strawberry, and we show that GS is a promising breeding strategy for polyploid species but that the actual advantage critically depends on the underlying genetic architecture. Source code, examples and a complete manual are freely available in GitHub https://github.com/lauzingaretti/pSBVB.

Genomic selection (GS) (Meuwissen *et al.* 2001) is the breeding strategy consisting in predicting future performance using DNA information from the whole genome, typically SNPs (single nucleotide polymorphisms). It relies on genome wide linkage disequilibrium (LD) between markers and the causal mutations, without the need to identify them. Due to dramatic reduction in genotyping costs, GS is becoming standard in many animal and plant breeding schemes, replacing or complementing traditional methods based solely on pedigree information. So far, GS has been mainly applied to diploid species. Yet, polyploidy is a very common phenomenon in evolution and include numerous species of interest (*e.g.*, strawberry, potato, wheat). Traditionally, polyploid species have been classified into autopolyploids, caused by one or more genome duplication events in a single species, and allopolyploids, the result of hybridization between closely related species (Stebbins 1947). The impact of GS on either auto- or allo-polyploid species breeding, however, remains largely unexplored.

In principle, the application of GS in polyploid species can have a positive impact in the rates of genetic gain through improved accuracy of predicted breeding values and/or reduction of generation intervals (Slater *et al.* 2016; Bassi *et al.* 2016; Sverrisdóttir *et al.* 2017; Gezan *et al.* 2017; Enciso-Rodriguez *et al.* 2018). However, the complex genetic structure of polyploids has delayed the availability of genome-wide genotyping SNP arrays that are needed for GS. Polyploid SNP detection can be challenging due to a high similarity between homologous and homeologous sequences, which generates complications to differentiate true SNPs from nuisance paralogous variants (Bassil *et al.* 2015; Clevenger and Ozias-Akins 2015).

Further, accurate genotyping is also important but becomes more complex as ploidy level increases. Several tools to perform genotype estimation from SNP array platforms are already available (Voorrips *et al.* 2011; Voorrips and Gort 2018; Schmitz Carley *et al.* 2017; Blischak *et al.* 2017). However, the arising of Next Generation Sequencing technologies requires of new tools adapted for this type of data, which are also being developed (Bourke *et al.* 2018; Meirmans *et al.* 2018; Gerard *et al.* 2018).

Computer simulation is a fundamental tool to evaluate alternative breeding schemes, since it allows the exploration of a wide range of hypothesis at no cost and can help to interpret the outcome of selection in complex situations. In this regard, numerous simulation tools have been developed such as easyPOP (Balloux 2001), simuPOP (Peng and Kimmel 2005; Peng and Amos 2008), forqS (Kessner and Novembre 2014) Slim (Messer 2013), PedigreeSim (Voorrips and Maliepaard 2012) among others. However, simulation approaches may not be straightforward to interpret owing to unknowns on the genetic architecture, among other factors. These problems are exacerbated in polyploid species and, to the best of our knowledge, only simuPOP and PedigreeSim allow polyploids organisms. simuPOP is not developed to compare breeding schemes, whereas PedigreeSim does not directly generate phenotypes nor produces genomic relationship matrices.

Here we present a flexible simulation tool for complex phenotypes adapted to polyploids and we propose several approaches to compute the molecular relationship matrix in polyploids. The software is an extension of Sequence-Based Virtual Breeding (SBVB, Pérez-Enciso *et al.* 2017), called pSBVB. This tool employs complete or partial genome data as input and simulates new genomes by gene dropping. We illustrate the software with data from two economically important polyploid species: potato, an autopolyploid, and strawberry, an allopolyploid.

## Methods

### Polyploid sequence based virtual breeding (pSBVB)

pSBVB is a modification of SBVB software (Pérez-Enciso *et al.* 2017) that allows simulating genotypes and phenotypes of an arbitrary genetic complexity in polyploids. Compared to SBVB designed for diploid organisms only, pSBVB enables simulating meiosis in autopolyploid or allopolyploid species (see below). It takes ploidy into account to generate the phenotypes and incorporates several options to compute the molecular relationship matrix that are pertinent to polyploids, as described below.

### Software algorithm

As input, pSBVB needs genotypes in vcf format (https://samtools.github.io) or a text file with genotypes coded to 0 up to *h* (where *h* is the ploidy level). For diploids, the vcf genotype format is of the kind 0/0, 0/1, and 1/1 for the three possible genotypes in a biallelic SNP. The polyploid vcf format is an extension of the type 0/0/0/0, 0/0/0/1 and so on in the case of an unphased tetraploid genotypes. Phased genotypes are represented by vertical bars, (*e.g.*, genotype 0|0|0|1 is different from 1|0|0|0) . No missing values are allowed. Phased genotypes are needed in pSBVB to identify which chromosomes are passed to offspring. A number of accurate phasing algorithms for diploids are available such as beagle (Browning and Browning 2007) or minimac (Howie *et al.* 2012). For polyploids, several approaches are also developed (He *et al.* 2018; Shen *et al.* 2016), but their accuracy has not been completely validated and seems critically dependent on ploidy level. If phase is unknown, pSBVB randomly generates a phase configuration. Further, linkage disequilibrium can be obtained by generating an individual genome out of a random pedigree starting with the founders’ genotypes. To do that, pSBVB incorporates the option ‘EXPAND_BASEPOP’, which generates additional founders’ by randomly crossing the available ones and random breeding for a pre-specified number of generations (see SBVB manual, https://lauzingaretti.GitHub.io/pSBVB/). A list with QTNs (Quantitative Traits Nucleotides) positions, a list of SNP positions to be used for GS, a pedigree file and a parameter file are also necessary. The pedigree file is used to perform the gene dropping simulation, *i.e.*, genotypes’ of the descendants along the pedigree are generated following Mendelian rules and a pre specified pairing rate between homologous and homoelogous pairs; for autopolyploids, pairing is at random. While performing gene dropping, pSBVB stores only the recombination breakpoints, which results in an efficient algorithm to recover marker genotypes and phenotypes.

pSBVB is very flexible in terms of the genetic architectures; it can simulate any number of traits with their specific QTNs and allelic effects. QTNs effects can be specified in a file or sampled from gamma, normal, or uniform distributions. In contrast to SBVB, though, pSBVB does not allow for epistasis. The Figure 2 shows a general representation of the pSBVB software, as well as screen shots.

As output, pSBVB produces phenotype and marker data of individuals obtained from the pedigree-based gene-dropping procedure. In addition, pSBVB can also compute molecular relationship matrices G using predefined marker subsets (*e.g.*, a genotyping array) or the whole sequence. For polyploids, G is computed by default from:

$$G=\frac{\left(M-hp\right){\left(M-hp\right)}^{T}}{hp{\left(1-p\right)}^{T}}$$ (1)

where M is a n×m matrix with elements containing the number of copies of the alternative allele for $i_{th}$ individual (i=1..n) and $j_{th}$ SNP (j=1..m), and *p* is a *m*-dimension vector with marker allele frequencies. Note that Equation 1 reduces to the standard formula in the case of diploidy (h=2) (VanRaden 2008).

Assessing the genotype for polyploids can be inferred from fluorescence intensity in SNP arrays or from read count in sequence data (Bourke *et al.* 2018) but may not be as accurate as for diploid organisms, specially at high ploidy levels. If genotyping is not accurate, a simple alternative is assuming that only one full homozygous can be distinguished for the rest of genotypes, *i.e.*, that a given marker allele behaves as fully dominant. To accommodate this, pSBVB allows computing a modified $G^{*}$ where element $m_{ij}$ is coded as 0 if all alleles are 0 and 1 otherwise. This is specified with the MIMIC_HAPLOID statement in the parameter file. The software also incorporates a ’MIMIC_DIPLOID’ option, which assumes only presence or absence of the alternative allele can be ascertained for genotype values higher than 2. In summary, the software is able to generate three G matrices:

- **Default option:** The true genotype, *i.e.*, number of copies of the alternative allele, is known without error ($G_{T}$). In this approach M (Equation 1) has elements varying between 0 to *h*.
- **MIMIC_DIPLOID option:** Only 0, 1 and 2 or more copies of a given allele can be distinguished. In this case, all genotypes with values larger than 2 area assigned a value ’2’, thus M (Equation 1) has elements ranging between 0 and 2 and ploidy is set to 2.
- **MIMIC_HAPLOID option:** It considers that only one full homozygous can be distinguished for the rest of genotypes, then M (Equation 1) has elements ranging between 0 and 1 and ploidy is set to 1.

### Modeling meiosis in polyploids

Autopolyploids species have polysomic inheritance where homologous and homeologous chromosomes are randomly paired during meiosis. In contrast, most of allopolyploids have disomic inheritance, resulting from preferential pairing between homologous chromosomes. However, there is a continuum between both extreme meiotic behaviors that can be modeled by preferential pairing factor (*θ*), which expresses the increased probability of pairing between homologous chromosomes (Bourke *et al.* 2017). In a generic case with $\frac{h}{2}$ sub-genomes, where *h* is the ploidy level, there are $\left(\begin{array}{c} h\\ 2 \end{array}\right)=\frac{h\left(h-1\right)}{2}$ possible paring combinations between all the chromosomes. pSBVB allows modeling meiotic pairing via a recombination h×h matrix:

$$R=\left(\begin{array}{ccccc} 0 & \frac{1}{h-1}+\theta_{12} & \frac{1}{h-1}+\theta_{13} & \ldots & \frac{1}{h-1}+\theta_{1h}\\ \frac{1}{h-1}+\theta_{12} & 0 & \frac{1}{h-1}+\theta_{23} & \ldots & \frac{1}{h-1}+\theta_{2h}\\ \frac{1}{h-1}+\theta_{13} & \frac{1}{h-1}+\theta_{23} & 0 & \ldots & \frac{1}{h-1}+\theta_{3h}\\ \ldots & \ldots & \ldots & \ldots & \ldots \\ \frac{1}{h-1}+\theta_{1h} & \frac{1}{h-1}+\theta_{2h} & \frac{1}{h-1}+\theta_{3h} & \ldots & 0 \end{array}\right)$$ (2)

where $\frac{1}{h-1}+\theta_{ij}$ ∀ i,j in Equation 2 represents the probability of pairing between *i* and *j* chromosomes, assuming chromosomes (1,2), (3,4), (h−1,h) are the homologous pairs.

For example, the matrix for a strict auto-tetraploid is:

$$R=\left(\begin{array}{cccc} 0 & 1/3 & 1/3 & 1/3\\ 1/3 & 0 & 1/3 & 1/3\\ 1/3 & 1/3 & 0 & 1/3\\ 1/3 & 1/3 & 1/3 & 0 \end{array}\right)$$ (3)

And for a strict allopolyploid would be:

$$R=\left(\begin{array}{cccc} 0 & 1 & 0 & 0\\ 1 & 0 & 0 & 0\\ 0 & 0 & 0 & 1\\ 0 & 0 & 1 & 0 \end{array}\right)$$ (4)

Internally, pSBVB assumes that the order of chromosomes in this matrix is the same as in the genotype alleles from the vcf file.

### Phenotype simulation

**Figure 1:**
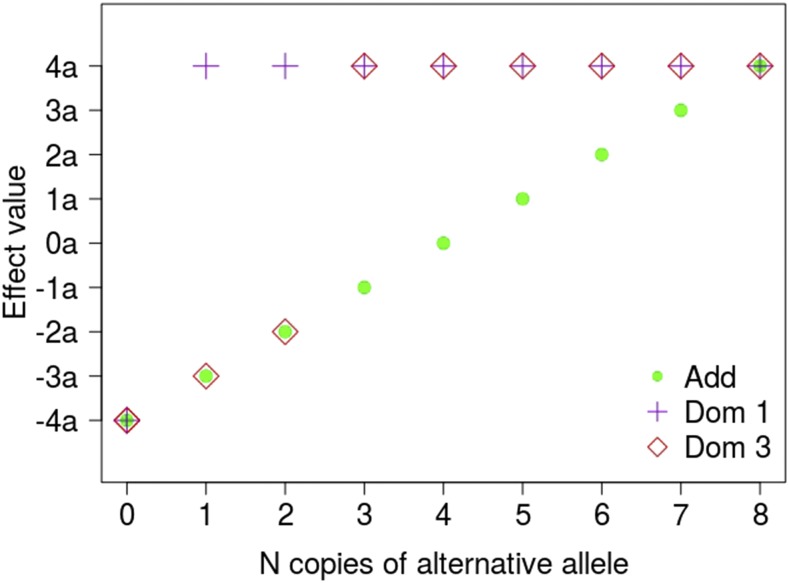
Additive and dominance modeling in polyploids used by pSBVB. The figure represents three possible genic actions in an octoploid. Under a strict additive action (∘), phenotype is expected to increase in an *a* unit per copy of the alternative allele, note that a can be negative or positive. Under a dominance scheme, and φ=1, phenotype is expected to be the same for any heterozygous genotype (+) (Dom 1), whereas under dominance and φ=3 (Dom 3), the genotype is expected to be the same for all heterozygous with more than 3 copies to alternative allele (◇).

In a diploid organism, the phenotype for $i_{th}$ individual can be simulated from:

$$y_{i}=\mu +\overset{Q}{\underset{j=1}{\sum }}\gamma_{ij}a_{j}+\overset{Q}{\underset{j=1}{\sum }}\delta_{ij}d_{j}+\epsilon_{i}$$ (5)

where *μ* is the general mean, $a_{j}$ is the additive effect of $j_{th}$ SNP, that is, half the expected difference between homozygous genotypes, $\gamma_{ij}$ takes values −1, 0 and 1 for homozygous, heterozygous and alternative homozygous genotypes, respectively, $d_{j}$ is the dominance effect of $j_{th}$ SNP, and $\delta_{ij}$ takes value 1 if the genotype is heterozygous, 0 otherwise, and $\epsilon_{i}$ is a normal residual of the *i*- observation. For polyploids, the equivalent equation can be expressed as:

$$y_{i}=\mu +\overset{Q}{\underset{j=1}{\sum }}\eta_{ij}a_{j}+\overset{Q}{\underset{j=1}{\sum }}\varphi_{ij}d_{j}+\epsilon_{i}$$ (6)

where $\eta_{ij}$ is the number of copies of the alternative allele (coded say as 1) minus half the ploidy (h/2) for $j_{th}$ SNP and $i_{th}$ individual, and $a_{j}$ is therefore the expected change in phenotype per copy of allele ’1’ in the $j_{th}$ SNP. In polyploids, as many dominance coefficients as ploidy level (*h*) minus 1 can technically be defined. However, this results in an over-parameterized model that is of no practical use. Here instead we define the $\varphi_{ij}$ parameter as the minimum number of copies of allele 1 such that the expected phenotype is $d_{j}$. In our modeling, all genotypes with number of copies over $\varphi_{ij}$ have the same expected phenotype. See Figure 1 for a graphical representation. By default, pSBVB takes $\varphi_{ij}=1$.

**Figure 2:**
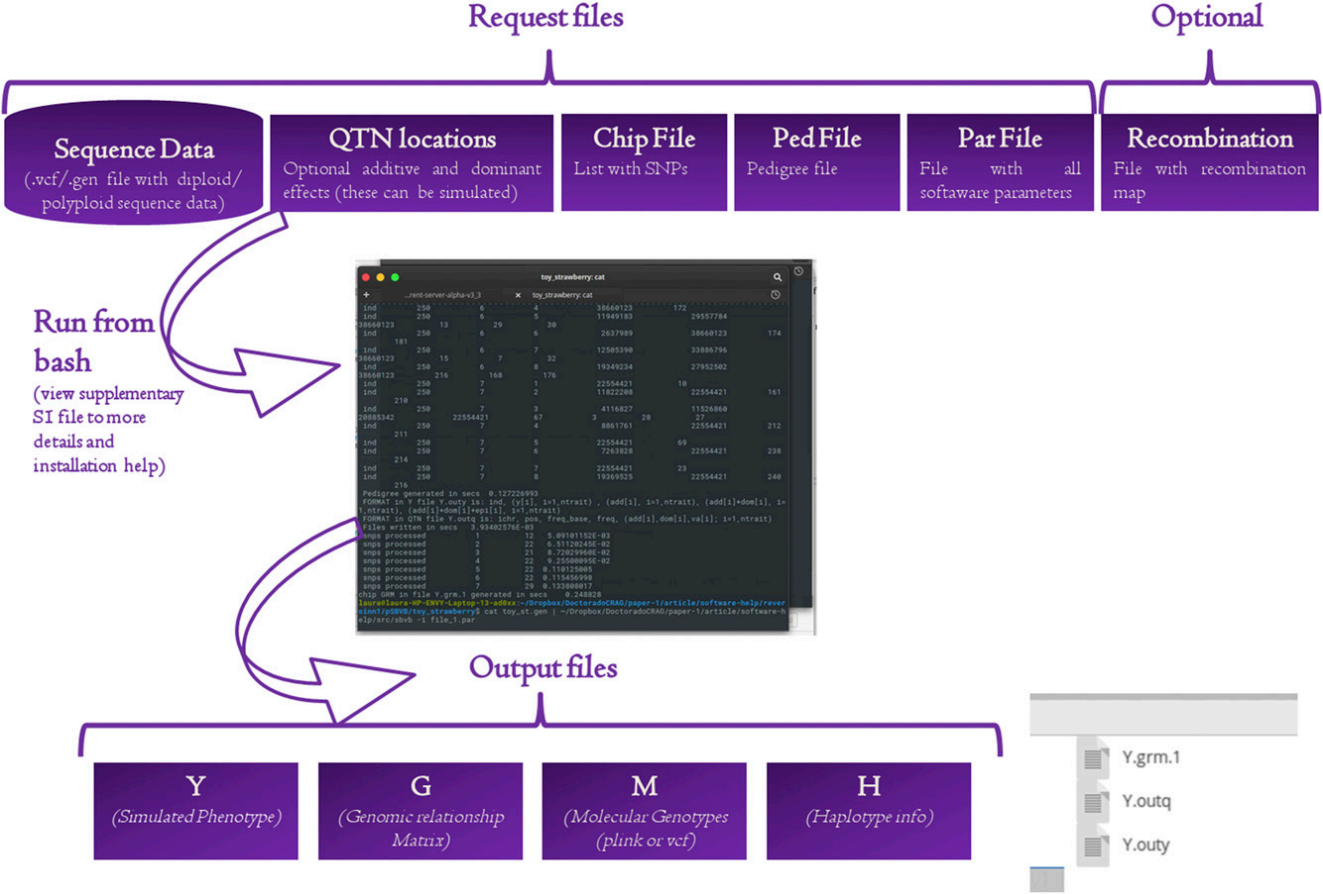
General representation of pSBVB software. As input, the software reads the vcf file containing all phased SNPs from founder haplotypes. Additional files specify the genetic architecture (it may include additive and dominant effects), the lists of SNPs (each corresponding to one genotyping array and/or complete sequence), and the recombination map for each sex and genome location (optional). pSBVB then performs gene dropping following a predetermined pedigree, generating phenotypes and true genotypes (Y), genomic relationship matrices (G, one per SNP list), and genotypes for each individual in the pedigree and for each SNP list in Plink or generic format, an optional file containing haplotype information that allows quick restart of the program, and information on QTN contribution to variance. Genomic Relationship Matrix can be computed using several options (see main text). As output, the software provides genomic relationship matrix (Y.grm.1), QTN’s effects (y.outq) and simulated phenotypes (y.outy).

### Statistical model for Genomic prediction

There are currently numerous statistical methods that address the large p small n problem and use genome-wide markers to predict breeding values (*e.g.*, de los Campos *et al.* 2009, Hayes *et al.* 2009). pSBVB does not compute genomic breeding values but can produce genomic relationship matrices suitable to obtain GBLUP (VanRaden 2008), as detailed above. Otherwise, pSBVB outputs genotypes of all or a subset of markers and any desired GS algorithm can be applied. R scripts are provided in GitHub that performs GBLUP.

### Data Availability

The source codes and the documented functions are distributed from GitHub: https://github.com/lauzingaretti/pSBVB. The manual includes a full tutorial of all functions at the program and a user guide with the installation guidelines and examples to simulate polyploid organisms. The software is accompanied by R scripts(R Core Team 2017) to generate a pedigree file, cmpute the numerator relationship matrix, perform GBLUP (VanRaden 2008) or assess predictive ability (PA). Examples showing the software capabilities with alternative parameter options are also available.

## Results

In order to illustrate the software capabilities, we have used dataset from two polyploids species: autoplyploid potato (*Solanum tuberosum*, 2n=4x=48) and allopolyploid strawberry (*Fragaria* x *ananassa* 2n=8x=56).

### Potato genotypes

The availability of an 8,300 SNP array has allowed the development of GS studies in potato, one of the most important crops worldwide (*e.g.*, Sverrisdóttir *et al.* 2017, Enciso-Rodriguez *et al.* 2018). To illustrate our tool, here we used a subset of 407 SNPs and 150 individuals from Enciso-Rodriguez *et al.* (2018). SNP positions were obtained from Rosyara *et al.* (2016). We used these genotypes to generate a vcf file where genotypes were coded between 0 and 4 (the potato ploidy level), phases were randomly generated.

Next, to generate linkage disequilibrium in the randomly phased dataset, we included additional dummy founders using the “EXPAND_BASEPOP” statement in the parameter file (see reference manual, https://lauzingaretti.github.io/pSBVB/). With this option, new base population individuals are obtained via randomly generated pedigrees. A new base population with 100 founders was obtained. The total pedigree size was 700, with 250 founders was obtained. The total pedigree size was 700, including 250 founders (150 initial individuals and the 100 new base population individuals) and four generations with 100, 100, 100 and 150 observations, respectively.

**Figure 3:**
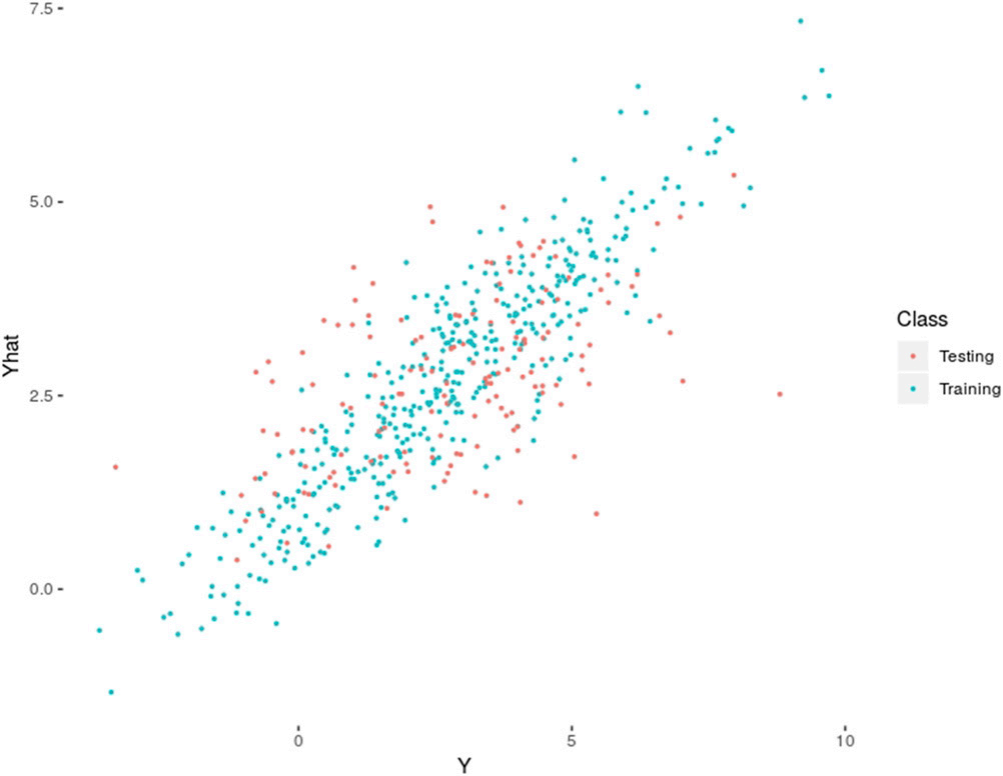
Predicted breeding values from GS model in the simulated potato dataset: correlations between observed and predicted values from training and testing populations were 0.91 and 0.52, respectively.

Phenotypes were simulated using 140 randomly chosen QTNs and heritability ($h^{2}$) was set to 0.5. As numerous studies suggest that allele distribution is highly leptokurtic (García-Dorado *et al.* 1998; Eyre-Walker and Keightley 2007) with many near-zero effects and a few large effects, we used a gamma Γ(α=0.2,β=5) distribution to simulate additive effects as in Caballero *et al.* (2015). G matrix was computed assuming that all markers are known without error, since the potato chip ensures that the true genotype can be obtained. Finally, to ilustrate GS performance, which was assessed removing the 150 individuals from the last generation and computing the correlation between predicted and observed phenotypes of these 150 individuals. Figure 3 plots the observed *vs.* predicted phenotypes in training (400 individuals) and test (150 individuals) population. In this example, PA was reasonably high (ρ=0.52), and illustrates that reasonable accuracies can be obtained even with small population sizes provided linkage disequilibrium and $h^{2}$ are relatively high.

The pedigree and the numerator relationship matrix files were generated using the pedigree.R and RelationshipMatrix.R functions, respectively; breeding values were predicted with GBLUP using GBlupFunction.R script. The whole source code and scripts to run this example are available at GibHub site.

### Application to strawberry GBS data

We also applied our program to octoploid strawberry *F*. x *ananassa*. In the absence of a reasonable number of strawberry sequenced genomes, we used unpublished data obtained with GBS (Genotyping by Sequencing) from 47 strawberry cultivars. Genotype-by-Sequencing libraries were prepared by Heartland Plant Innovations (http://www.heartlandinnovations.com/). Samples were multiplexed and sequenced 92 cycles on the Illumina MiSeq at the Oklahoma Medical Research Foundation. Data quality was checked by FASTQC (http://www.bioinformatics.babraham.ac.uk/projects/fastqc/). To obtain reasonably realistic genotypes based on these data, we applied the following pipeline. GBS reads were aligned against *Fragaria vesca* (diploid strawberry) reference genome (F. vesca-genome.v2.0.a1), bam files were filtered setting minimum base and mapping qualities to 37 and 20, respectively, and parsed with snape (https://github.com/EmanueleRaineri/snape-pooled, Raineri *et al.* 2012), a SNP caller developed for pools.

This software requires as input the number of diploid individuals in the pool, which was set to four. Polymorphic positions with fewer than 20 high quality reads were removed, as well as those where more than 60% of the cultivars were not covered. Logically, only allele counts 0, 1, to 8 are allowed in an octoploid genome SNP, whereas the number of reads per position follows a quasi-continuous distribution. To convert number of reads to genotype score, we computed the fraction of alternative allele reads divided by the total number of reads (*f*) and inferred its genotype from the nearest possible integer to f×8. This was done for each SNP and cultivar. Missing genotypes were sampled according to the genotype frequency in the non-missing positions for that SNP. We assumed independence to perform the assignations. A total of 50,609 variant positions were obtained (5779, 7985, 7328, 6362, 8282, 9012, 5862 in linkage groups GL1, GL2, GL3, GL4, GL5, GL6 and GL7, respectively). These markers were used as genetic file input for the program. Among those SNPs, ∼ 36%, 37%, 14% and 13% variants were classified as segregating in 1, 2, 3 or all sub- genomes: *2x*, *4x*, *6x* and *8x*, respectively.

Strawberry breeding programs are based on evaluating crosses between elite lines. Traditional crop breeding is expensive and time consuming and GS can accelerate strawberry improvement if only a subset of these crosses were fully tested in the field. To mimic this scenario, we generated a pedigree file with five generations of intercrossing starting with the 53 base population lines. Each generation was made up of 100 lines. In the last generation, 1000 crosses with unknown phenotype were generated from the 100 current parental lines. As measure of predictive accuracy, we computed the correlation between observed and predicted phenotypes of the 1000 crosses, when the phenotypes from these 1000 crosses were removed. One hundred replicates were run per case.

To simulate the phenotypes, we considered a range of genetic architectures with a focus on sugar content:

• Random QTNs in sugar associated Pathways (RQP): 100 SNPs were randomly chosen as causal among the SNPs in the sugar pathway associated genes ± 10 kb.
• Diploid QTNs in sugar associated Pathways (DQP): 100 SNPs were randomly chosen as causal among the diploid SNPs in the sugar pathway associated genes ± 10 kb.
• Random QTNs Genome-wide chosen (RQG): 100 SNPs were randomly chosen as causal among all detected SNPs.

In the first two architectures, we aimed at mimicking a trait of economic interest such as sucrose content. The gene information was obtained from FragariaCyc (http://pathways.cgrb.oregonstate.edu, Naithani *et al.* 2016). In total, there were 159 genes containing 499 SNPs associated with these pathways. Within each of the three architectures, phenotypes were simulated according to two extreme gene actions: fully additive and complete dominance (φ=1, Figure 1). Heritability was set to 0.5.

For each architecture, phenotypes were simulated according to two extreme gene actions: fully additive and complete dominance. In the dominant approach, we set Γ(α=0.2,β=5) (Figure 1). Each phenotype was generated from its genotypic value adding an environmental effect, where was adjusted such that heritability was $h^{2}=0.5$.

**Figure 4:**
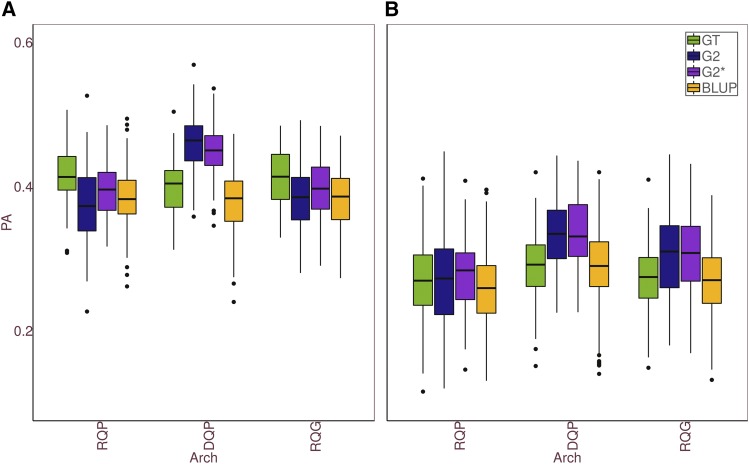
Predictive Ability ($PA=cor\left(y,\hat{y}\right)$) of GBPLUP and P−BLUP models for each of the three genetic architectures considered in strawberry simulated dataset: random QTNs in sugar associated pathways (RQP), diploid QTNs in sugar associated pathways (DQP) and genome-wide chosen (RQG) and each of the three GBLUP models. Three GBLUP models were compared: In GT, genetic matrix G was computed assuming SNP allele frequencies were known without error; in G2, only diploid SNPs were used, and genotypes were known without error; and in $G2^{*}$, G Genomic relationship matrix is computed assuming than only presence or absence of the alternative allele could be known for the remaining, *i.e.*, although the organism was polyploid, Genomic relationship matrix is computed assuming than only presence or absence of the alternative allele can be ascertained. (a) additive architecture; (b) dominant architecture.

Simulated PAs are in Figure 4. We estimated the PA using the following matrices:

- GT: The true genotype, *i.e.*, number of copies of the alternative allele, was known without error and all SNPs were used. In this approach Equation M (Equation 1) has elements varying between 0 and 8.
- G2: Only diploid SNPs were used, and genotypes were known without error. M (Equation 1) has elements ranging between 0 and 2.
- $G2^{\text{*}}$: All SNPs were employed but only genotypes of diploid SNPs were known without error, whereas for the remaining, although the organism was polyploid, Genomic matrix is computed mimic diploid. M (Equation 1) has elements ranging between 0 and 2.
- **Numerator Relationship Matrix (P-BLUP)**: The breeding values were predicted using the pedigree relationship matrix.

Figure 4 shows the obtained accuracies across genetic architectures and for each evaluation method. Overall, these results indicate that performance of GS in polyploids may critically depend on the underlying genetic architecture. Unsurprisingly, accuracy also drops when dominance exists compared to the additive scenarios. Several additional observations of interest can be drawn from Figure 4. First, there were no differences in the ranking of methods irrespective of whether QTN were scattered throughout the genome (RQG) or localized in given segments (RQP). This was observed for both additive and dominant architectures. Second, using the true genotype values to build **G** (**GT**) did not always outperform the rest of GBLUP methods considered. In fact, this was observed only when the architecture was fully additive and the QTNs were segregating in more than one homeolog group. In these cases, **GT**-BLUP was ∼4−8% better than G2-BLUP or G2*-BLUP. **G2**, which employs only diploid SNPs, should be preferred to **GT**-BLUP only if QTNs are exclusively diploid. A relevant result is that G2* -BLUP, which treats markers as dominant, was a quite robust strategy, in particular with complete dominance and with the exception of DQP scenario (*i.e.*, when all QTNs were diploid).

Finally, note that the advantage of GBLUP over P-BLUP is not always guaranteed. At least in the breeding scenario analyzed here, **G2**-BLUP might actually perform worse than **P-BLUP** when QTNs segregate randomly (RQP and RQG) and genic action is additive. If true SNP genotypes could be known without error (**GT**), the increase in accuracy compared to **P-BLUP** would vary between ∼7% and 18%. As for using G2*-BLUP, increase in accuracy was between ∼3% and ∼16% across all cases examined here. The advantage, though, would diminish if genic action were additive and QTN would segregate in all homologous.

The genetic file used as input includes 1500 SNPs from the whole *vcf* file. More examples combining a set of different parameters (additive and dominance effects, Genetic Matrix calculation, pedigree and Genomic Relationship Generation, among others) are available on GitHub.

## Discussion

Certainly, polyploid sequence data will be increasingly available, which will be used to achieve a better understanding of complex trait genetics and to optimize GS strategies. To help in the latter task, here we have developed an extension of SBVB software (pSBVB) that feeds from real sequence data of polyploid organisms. It uses efficient forward algorithms and allows simulating meiosis in polyploid species, suited for both auto and allopolyploid organisms. Further, pSBVB generalizes genetic modeling in polyploids to generate phenotypes and incorporates several options to compute predefined molecular relationship matrices that are specific to polyploid organisms. Note though that, since pSBVB can print the whole SNP dataset, any custom-made **G** can be computed and any alternative GS method can be evaluated. There are some limitations though. An important one is that epistasis cannot be modeled in pSBVB -in contrast to the diploid version (SBVB)- but this limitation stems from the lack of realistic modeling on epistasis for polyploids rather than out of computational constraints.

To the best of our knowledge, there are no simulation tools that allow estimate genetic matrix in polyploids organisms with a range of options like the one described here. Among the available forward-time simulation tools, only simuPOP (Peng and Kimmel 2005; Peng and Amos 2008) and PedigreeSim (Voorrips and Maliepaard 2012) consider polyploids. Compared to simuPOP, pSBVB allows simulating both auto and allo-polyploids organisms, accepting as input a recombination matrix between homeolog groups. PedigreeSim is not specifically designed for GS and is not able to simulate complex genetic architectures and relationships matrices as pSBVB. A further outcome of our work is the proposal of several G matrices that are robust to genotype misspecification, an important problem in polyploids (Bourke *et al.* 2017).

To conclude, we have developed a flexible GS simulation tool capable of using real sequence data from polyploids. We show the tool capabilities using potato and strawberry real datasets. With potato genotypes, we illustrate how new base population individuals can be generated and show that accuracy can be relatively high even with modest population sizes. Among the molecular relationship matrices proposed, assuming that only diploid genotypes can be identified seems overall a good compromise in terms of performance, at least in strawberry data. Our study suggests that GS may increase response to selection compared to **P-BLUP**, but this will depend on the true genetic architecture of the trait, as also shown by Gezan *et al.* (2017) with real strawberry data. We urge advancing on the quantitative and molecular dissection of complex traits in polyploids, which should provide important parameters such as prevalent genic action or number of segregating homeolog groups, in order to design optimum GS breeding schemes for these species.

## References

[bib1] Balloux F., 2001 EASYPOP (Version 1.7): A Computer Program for Population Genetics Simulations. J. Hered. 92: 301–302. 10.1093/jhered/92.3.301 11447253

[bib2] Bassi F. M. Bentley A. R. Charmet G. Ortiz R. Crossa J., 2016 Breeding schemes for the implementation of genomic selection in wheat (triticum spp.). Plant Sci. 242: 23–36. 10.1016/j.plantsci.2015.08.021 26566822

[bib3] Bassil N. V. Davis T. M. Zhang H. Ficklin S. Mittmann M., 2015 Development and preliminary evaluation of a 90 K Axiom SNP array for the allo-octoploid cultivated strawberry Fragaria × ananassa. BMC Genomics 16: 155 10.1186/s12864-015-1310-1 25886969 PMC4374422

[bib4] Blischak P. D. Kubatko L. S. Wolfe A. D., 2017 SNP genotyping and parameter estimation in polyploids using low-coverage sequencing data. Bioinformatics 34(3): 407–415. 10.1093/bioinformatics/btx587 29028881

[bib5] Bourke P. M. Arens P. Voorrips R. E. Esselink G. D. Koning-Boucoiran C. F., 2017 Partial preferential chromosome pairing is genotype dependent in tetraploid rose. Plant J. 90: 330–343. 10.1111/tpj.13496 28142191

[bib6] Bourke P. M. Voorrips R. E. Visser R. G. F. Maliepaard C., 2018 Tools for Genetic Studies in Experimental Populations of Polyploids. Front. Plant Sci. 9: 513 10.3389/fpls.2018.00513 29720992 PMC5915555

[bib7] Browning S. R. Browning B. L., 2007 Rapid and accurate haplotype phasing and missing-data inference for whole-genome association studies by use of localized haplotype clustering. Am. J. Hum. Genet. 81: 1084–1097. 10.1086/521987 17924348 PMC2265661

[bib8] Caballero A. Tenesa A. Keightley P. D., 2015 The Nature of Genetic Variation for Complex Traits Revealed by GWAS and Regional Heritability Mapping Analyses. Genetics 201: 1601–1613. 10.1534/genetics.115.177220 26482794 PMC4676519

[bib9] Clevenger J. P. Ozias-Akins P., 2015 SWEEP: A Tool for Filtering High-Quality SNPs in Polyploid Crops. G3: Genes, Genomes. Genetics 5: 1797–1803. 10.1534/g3.115.019703 PMC4555216 26153076

[bib10] e los Campos G. Naya H. Gianola D. Crossa J. Legarra A. Manfredi E., 2009 Predicting Quantitative Traits with Regression Models for Dense Molecular Markers and Pedigree. Genetics 182: 375–385. 10.1534/genetics.109.101501 19293140 PMC2674834

[bib12] Enciso-Rodriguez F. Douches D. Lopez-Cruz M. Coombs J. de los Campos G., 2018 Genomic Selection for Late Blight and Common Scab Resistance in Tetraploid Potato (Solanum tuberosum). G3: Genes, Genomes, Genetics, 8(7): 2471–2481. 10.1534/g3.118.200273 29794167 PMC6027896

[bib13] Eyre-Walker A. Keightley P. D., 2007 The distribution of fitness effects of new mutations. Nat. Rev. Genet. 8: 610–618. 10.1038/nrg2146 17637733

[bib14] García-Dorado A. Monedero J. L. López-Fanjul C., 1998 The mutation rate and the distribution of mutational effects of viability and fitness in Drosophila melanogaster. Genetica 102–103: 255–265. 10.1023/A:1017086909282 9720284

[bib15] Gerard D. Ferrão L. F. V. Garcia A. A. F. Stephens M., 2018 Genotyping polyploids from messy sequencing data. Genetics, 210(3): 789–807. 10.1534/genetics.118.301468 PMC6218231 30185430

[bib16] Gezan S. A. Osorio L. F. Verma S. Whitaker V. M., 2017 An experimental validation of genomic selection in octoploid strawberry. Hortic. Res. 4: 16070 10.1038/hortres.2016.70 28090334 PMC5225750

[bib17] Hayes B. J. Bowman P. J. Chamberlain A. J. Goddard M. E., 2009 Invited review: Genomic selection in dairy cattle: Progress and challenges. J. Dairy Sci. 92: 433–443. 10.3168/jds.2008-1646 19164653

[bib18] He D. Saha S. Finkers R. Parida L., 2018 Efficient algorithms for polyploid haplotype phasing. BMC Genomics 19: 110 10.1186/s12864-018-4464-9 29764364 PMC5954289

[bib20] Howie B. Fuchsberger C. Stephens M. Marchini J. Abecasis G. R., 2012 Fast and accurate genotype imputation in genome-wide association studies through pre-phasing. Nat. Genet. 44: 955–959. 10.1038/ng.2354 22820512 PMC3696580

[bib21] Kessner D. Novembre J., 2014 forqs: forward-in-time simulation of recombination, quantitative traits and selection. Bioinformatics 30: 576–577. 10.1093/bioinformatics/btt712 24336146 PMC3928523

[bib22] Meirmans P. G. Liu S. van Tienderen P. H., 2018 The analysis of polyploid genetic data. J. Hered. 109: 283–296. 10.1093/jhered/esy006 29385510

[bib23] Messer P. W., 2013 SLiM: simulating evolution with selection and linkage. Genetics 194: 1037–1039. 10.1534/genetics.113.152181 23709637 PMC3730910

[bib24] Meuwissen T. H. E. Hayes B. J. Goddard M. E., 2001 Prediction of total genetic value using genome-wide dense marker maps. Genetics 157: 1819–1829. 11290733 10.1093/genetics/157.4.1819 PMC1461589

[bib25] Naithani S. Partipilo C. M. Raja R. Elser J. L. Jaiswal P., 2016 FragariaCyc: A Metabolic Pathway Database for Woodland Strawberry Fragaria vesca. Front. Plant Sci. 7: 1–10. 10.3389/fpls.2016.00242 26973684 PMC4777718

[bib26] Peng B. Amos C. I., 2008 Forward-time simulations of non-random mating populations using simuPOP. Bioinformatics 24: 1408–1409. 10.1093/bioinformatics/btn179 18417488 PMC2691961

[bib27] Peng B. Kimmel M., 2005 simuPOP: A forward-time population genetics simulation environment. Bioinformatics 21: 3686–3687. 10.1093/bioinformatics/bti584 16020469

[bib28] Pérez-Enciso M. Forneris N. de los Campos G. Legarra A., 2017 Evaluating Sequence-Based Genomic Prediction with an Efficient New Simulator. Genetics 205: 939–953. 10.1534/genetics.116.194878 27913617 PMC5289861

[bib29] R Core Team, 2017 R: A Language and Environment for Statistical Computing.

[bib30] Raineri E. Ferretti L. Esteve-Codina A. Nevado B. Heath S., 2012 SNP calling by sequencing pooled samples. BMC Bioinformatics 13: 239 10.1186/1471-2105-13-239 22992255 PMC3475117

[bib31] Rosyara U. R. De Jong W. S. Douches D. S. Endelman J. B., 2016 Software for genome-wide association studies in autopolyploids and its application to potato. Plant Genome 9: 1–10. 10.3835/plantgenome2015.08.0073 27898814

[bib32] Schmitz Carley C. A. Coombs J. J. Douches D. S. Bethke P. C. Palta J. P., 2017 Automated tetraploid genotype calling by hierarchical clustering. Theor. Appl. Genet. 130: 717–726. 10.1007/s00122-016-2845-5 28070610

[bib33] Shen J. Li Z. Chen J. Song Z. Zhou Z., 2016 Shesisplus, a toolset for genetic studies on polyploid species. Sci. Rep. 6: 24095 10.1038/srep24095 27048905 PMC4822172

[bib34] Slater A. T. Cogan N. O. Forster J. W. Hayes B. J. Daetwyler H. D., 2016 Improving genetic gain with genomic selection in autotetraploid potato. Plant Genome 9: 1–15. 10.3835/plantgenome2016.02.0021 27902807

[bib35] Stebbins G. L., 1947 Types of Polyploids: Their Classification and Significance. Adv. Genet. 1: 403–429. 10.1016/S0065-2660(08)60490-3 20259289

[bib36] Sverrisdóttir E. Byrne S. Sundmark E. H. R. Johnsen H. Ø. Kirk H. G., 2017 Genomic prediction of starch content and chipping quality in tetraploid potato using genotyping-by-sequencing. Theor. Appl. Genet. 130: 2091–2108. 10.1007/s00122-017-2944-y 28707250 PMC5606954

[bib37] VanRaden P., 2008 Efficient Methods to Compute Genomic Predictions. J. Dairy Sci. 91: 4414–4423. 10.3168/jds.2007-0980 18946147

[bib38] Voorrips, R. E. and G. Gort, 2018 *fitPoly: Genotype Calling for Bi-Allelic Marker Assays*. R package version 3.0.0.

[bib39] Voorrips R. E. Gort G. Vosman B., 2011 Genotype calling in tetraploid species from bi-allelic marker data using mixture models. BMC Bioinformatics 12: 172 10.1186/1471-2105-12-172 21595880 PMC3121645

[bib40] Voorrips R. E. Maliepaard C. A., 2012 The simulation of meiosis in diploid and tetraploid organisms using various genetic models. BMC Bioinformatics 13: 248 10.1186/1471-2105-13-248 23013469 PMC3542247

